# Supplementary material for: Cognition and Neuropsychological Changes at Altitude—A Systematic Review of Literature
Source: Brain Sci. 2022 Dec 19;12(12):1736. doi: 10.3390/brainsci12121736 (PMC9775937; doi:10.3390/brainsci12121736)
Supplement: Supplementary file 1 [file brainsci-12-01736-s001.zip › brainsci-2080249-supplementary.pdf]

Supplementary Materials

Table S1. STAR data reporting guidelines [35] assessed in the studies included in the current analysis.

Section	Parameters
Section 1: SETTING	1.1 Study location
	1.2 Setting
	1.3 Altitude
	1.4 Starting point of the ascent
	1.5 Maximum altitude reached
	1.6 Mode of ascent (active or passive)
Section 2: INDIVIDUAL FACTORS	2.1 Age
	2.2 Sex
	2.3 Pre-existing altitude exposure
	2.4 High-altitude native
	2.5 Pre-existing health conditions
	2.6 History of prior AMS
	2.7 History of prior HACE
	2.8 History of prior HAPE
	2.9 Pre-acclimatization
	2.10 Altitude of residence
Section 3: AMS & HACE	3.1 Headache
	3.2 Gastro-intestinal symptoms
	3.3 Fatique / weakness
	3.4 Dizziness / light-headedness
	3.5 Ataxia
	3.6 Change in mental status
	3.7 AVPU
	3.8 S_pO_2
	3.9 Time of fulfilling AMS definition – this suggests time of onset, not diagnosis
	3.10 Time of fulfilling HACE definition – same, needs to be consistent with text
	3.11 New Lake Louise AMS Score
Section 4: HAPE	4.1 Weakness / decreased exercise performance
	4.2 Dyspnea at rest
	4.3 Cough
	4.4 Tachypnea
	4.5 Orthopnea
	4.6 Pink frothy sputum
	4.7 Respiratory Rate
	4.8 Heart Rate
	4.9 S_pO_2
	4.10 Rales and wheezing
	4.11 Time of fulfilling HAPE definition – as above
Section 5: TREATMENT	5.1 List all drugs with generic names, dosages, mode of administration, dosage intervals and indication
	5.2 Supplemental oxygen
	5.3 Hyperbaric bag
	5.4 Descent

Legend.
AMS: Acute mountain sickness; AVPU: Acronym from "alert, verbal, pain, unresponsive"; HACE: High-altitude cerebral edema; HAPE: High-altitude pulmonary edema; S_pO_2: Oxygen saturation.

Table S2. List of articles and their findings of altitude impact on cognitive performance.

**First authors**	**Number of participants (sex)/Mean age of participants (Mean ± SD); profession if mentioned**	**Study protocol: Time of exposure, experimental condition, altitude profile, maximum altitude reached (Mode of ascent – active/passive) - Study protocol baseline or control group (CG), altitude (test event if named)**	**STAR core parameters**	**Timing of neuropsychological test administration under exposure (test event if named)/Duration of Test Battery**	**Test Battery: Neuropsychological tests (*Cognitive Domain*) + Further investigations that are not part of this review**	**Changes in cognitive performance ↔↑↓ based on significant results, p < 0.05 (test event if named)**
Abraini (1998) [42]	8 (M)/26.5 (range 24–37) y; climbers<br><br>Control group (CG) 8 (M)/24.5 (range 22–40) y	31-days, hypobaric chamber, sea level (SL) to 8,848 m above sea level (ASL)<br>-<br>6-days pre-acclimatization at 4,350 m (passive); 3x Pre baseline cognitive assessment (BCA) and 1x Post cognitive assessment (CA), each at SL<br><br>CG at SL	Section 1 2.1-2.3 2.9 Section 3 4.8, 4.9	7 assessments over 31 days between 4,500 m and 8,848 m/Not available (NA)	Pegboard-Psychomotor Test (*Psychomotor ability*) Number Ordination Rey's Test (*Mental efficacy - reasoning*) Visual Choice Reaction Time (*reaction time*)	<u>Pegboard-Psychomotor Test</u><br>↓ At 8,000 m (compared to CG), at 8,848 m and post exposure (compared to BCA)<br><u>Number Ordination</u><br>↑ Until 6,500 m (compared to BCA)<br>↓ At 8,000 & 8,848 m and post exposure (compared to CG)<br>↔ <u>Visual Choice Reaction Time</u>
Altbäcker (2019) [61]	12 (2 F)/32.72 (range 28–39) y; recreational climbers	43 min, breathing of hypoxic gas mixture via oronasal breathing mask equivalent to 5,500 m of altitude<br>-<br>Pre BCA under normoxia, Post CA	1.1-1.3 1.5 2.1-2.5 2.9, 2.10	After 23 min of hypoxic gas exposure/20 min	Modified CPT (*attention*) Number-Size Stroop Variant Task (*executive function*)<br>+<br>EEG recordings and ERP data analysis	<u>CPT</u><br>↔ Overall performance<br><u>Stroop Task</u><br>↔ Overall performance<br>↑ Efficiency for congruent and neutral stimuli
Asmaro (2013) [27]	35 (4 F)/NA (range 19-69 y); aviation industry employees	35 min, hypobaric chamber, 5 min at 7,620 m ≙ 25,000 ft following 30 min at 5,334 m ≙ 17,500 ft with intermittent breathing of O_2<br>-<br>Pre BCA and Post CA, altitude NA	1.1-1.5 2.1-2.3 3.6 5.2	3 assessments after entering chamber at 7,620 m ≙ 25,000 ft/5 min and two tests at 5,334 m ≙ 17,500 ft starting at the first and second half	Word-Color Stroop Task[1] (*executive function*) Digit Span Forward Task[1] (DST-F) (*short term memory*) DS Backward Task[1] (DST-B) (*working memory*) Trailmaking A Task[1] (*attention*) Trailmaking B Task[1] (*executive function*)	<u>Stroop Task</u><br>↓ Correct trials<br>↑ Faster reaction times at 7,620 m<br><u>DST-F</u> at 7,620 m & <u>DST-B</u> at 5,334 & 7,620 m<br>↓ Correct responses<br><u>Trailmaking A and B Task</u><br>↓ Slower completion times at 5,334 & 7,620 m

First authors	Number of participants (sex)/Mean age of participants (Mean ± SD); profession if mentioned	Study protocol: Time of exposure, experimental condition, altitude profile, maximum altitude reached (Mode of ascent – active/passive) - Study protocol baseline or control group (CG), altitude (test event if named)	STAR core parameters	Timing of neuropsychological test administration under exposure (test event if named)/Duration of Test Battery	Test Battery: Neuropsychological tests (*Cognitive Domain*) + Further investigations that are not part of this review	Changes in cognitive performance ↔↑↓ based on significant results, $p < 0.05$ (test event if named)
				of the 30-min exposure/NA		
Bjursten (2010) [46]	7 (NA)/NA (range 42-51 y); physicians (≥5 y of alpine experience)	5 days, field study, 1,115 m (NA) to 3,000 m (passive) to 4,554 m (active)<br>-<br>Pre-study: one night at 2,864 m (passive)<br>Familiarization session, Pre BCA and Post CA at 1,115 m	Section 1 2.1, 2.2, 2.6, 2.9, 2.10<br>Section 3 4.8, 4.9	3 assessments at 3,647 m (2), 4,554 m (3) and again at 3,647 m (4)/25 min	CNS Vital Signs[1]:<br>Verbal Memory Test (*memory*),<br>Visual Memory Test (*memory*),<br>Finger Tapping Test (psychomotor speed),<br>DSST (reaction time),<br>Word-Color Stroop Test (executive function),<br>Shifting Attention Test (executive function)	↔ <u>Verbal and Visual Memory Test</u>, <u>Finger Tapping Test</u>, <u>DSST</u>, <u>Word-Color Stroop Test</u> and <u>Shifting Attention Test</u><br><br>Negative correlations of the Lake Louise Scale & calculated cognitive domains<br>memory at 3,647 m (2)<br>Reaction time at 4,554 & 3,647 m (3 & 4)<br>Processing speed at 4,554 & 3,647 m (3 & 4)
Bonnon (1999) [47]	7 (3 F)/NA; occasional alpinists	30 days, field study, 3,500 m (passive) to 6,200 m (active)<br>-<br>BCA at 300 m (A1)<br>CG under normoxia	Section 1 2.1-2.3<br>Section 3 5.4	2 CAs day 2 at 3,500 m (A2),<br>day 17 at 5,400 m (A3)/NA	Cognitive Motor Task with Pocket Calculator (*Short term memory*)<br>+<br>Semidirected and subject-centered interview about mood states	↔ <u>Cognitive Motor Task</u> compared to CG
Chroboczek (2021) [62]	15 (M)/23.1 ± 2.1 y; physically active	30 min, breathing of hypoxic gas mixture via face mask equivalent to 3,500 m ≙ F_iO_2 13 %<br>-<br>Familiarization session, CA under normoxia, altitude NA	1.1-1.3<br>2.1-2.3<br>2.5	One assessment after 30 min of acute exposure/NA	Stroop Interference Test (*executive function*)<br>Stage 1. giving "names" of colors<br>Stage 2. "reading" color names<br>Stage 3. giving the name of the font color, each word is written with	↓ Slower time and time delta of stage 3 naming interference values
Davranche (2016) [24]	11 (M)/28 ± 8 y; regular recreational climbers	4 days, field study, 4,350 m ≙ 14,272 ft (passive)<br>-<br>Familiarization session, CA at SL	Section 1 2.1, 2.2, 2.5, 2.6<br>Section 3 4.8	3 assessments on the day of arrival (D0),	Simon Task (*excecutive function*)<br>Time Perception Task (*spatiotemporal Integration*)	<u>Simon Task</u><br>↓ Slower reaction times (D0)<br>↓ Decision errors twice as high over time of

First authors	Number of participants (sex)/Mean age of participants (Mean ± SD); profession if mentioned	Study protocol: Time of exposure, experimental condition, altitude profile, maximum altitude reached (Mode of ascent – active/passive) - Study protocol baseline or control group (CG), altitude (test event if named)	STAR core parameters	Timing of neuropsychological test administration under exposure (test event if named)/Duration of Test Battery	Test Battery: Neuropsychological tests (*Cognitive Domain*) + Further investigations that are not part of this review	Changes in cognitive performance ↔↑↓ based on significant results, $p < 0.05$ (test event if named)
				the second day (D2) and fourth day (D4)		altitude exposure with the congruent trials<br>↔ Decision errors with the incongruent trials<br>↓ Time Perception Task<br>Underestimating of durations
De Aquino Lemos (2012) [66]	10 (M)/24.9 ± 5.02 y	24 h, normobaric chamber, 433 mmHg ≙ 4,500 m ASL ≙ $F_iO_2$13,5 %<br>-<br>BCA at normoxic conditions, altitude NA	1.1-1.3<br>2.1-2.3<br>2.5<br>3.1, 3.2	One Assessment 24 hours after the start of exposure to hypoxia/NA	DST-F (*short term memory*)<br>DST-B (*executive function*)<br>Sequences of Numbers and Letters (*working memory*)<br>Corsi Blocks Forward (*short term memory*) and Backward (*working memory*)<br>Random Number Generation (*working memory*)<br>Stroop Color and Word Test (*attention*)<br>+<br>Polysomnography, EEG, Brunel Mood Scale questionnaire	↓ DST<br>Lower sum of the ranks, sum of direct order, sum of order, and span of direct order<br>↓ Sequence of Numbers and Letters Test<br>Lower number of correct answers<br>↓ Corsi Block Tests<br>Lower values for direct order, inverse order, sum of orders, and span of order inverse<br>↓ Random Number Generator Test<br>Higher index indicating worsening<br>↓ Stroop Color Test<br>Changes varying in expression over the three test stages with higher completion time, less correct answers and more errors
De Bels (2019) [76]	17 (M)/26.3 ± 8.1 y	4 h with ascent and descent, exposure time "on the top" 3 h 20 min, hypobaric chamber, reproduction of a cable car ride scheme and stay at a mountain top, 3,842 m<br>-	1.1-1.3<br>2.1, 2.2, 2.4, 2.5<br>4.8, 4.9	One assessment after at least 1 h of exposure/NA	PEBL:<br>Modified Math Processing Task (*Arithmetic Reasoning Problems*),<br>Perceptual Vigilance Task (*reaction time*),	↔ No change in performance in all three tests

First authors	Number of participants (sex)/Mean age of participants (Mean ± SD); profession if mentioned	Study protocol: Time of exposure, experimental condition, altitude profile, maximum altitude reached (Mode of ascent – active/passive) - Study protocol baseline or control group (CG), altitude (test event if named)	STAR core parameters	Timing of neuropsychological test administration under exposure (test event if named)/Duration of Test Battery	Test Battery: Neuropsychological tests (*Cognitive Domain*) + Further investigations that are not part of this review	Changes in cognitive performance ↔↑↓ based on significant results, $p < 0.05$ (test event if named)
		Pre-ascent BCA and Post-descent CA at SL			Time Wall Estimation Task (*spatiotemporal integration*) + Critical flicker fusion frequency	
Dykiert (2010) [48]	10 (4 F)/21.5 ± 1.4 (range 19-24) y	20 days, field study, from 1,992 m to 3,271 m (passive) then with 1.5 m/min up to 5,565 m (active) ASL - 4 x CA at 76 – 86 m ASL prior to expedition	Section 1 2.1, 2.2, 2.3 Section 3 4.8	21 assessments, approx. daily/NA	Four-choice RT (*attention*)	↓ <u>Four-choice RT</u> Increased reaction times above 4,000 m revealed in linear mixed modeling
Falla (2021) [49]	36 (18 F)/ 27.3 ± 4.1 (range 22–40) y; health care personnel	3 days, field study, From 1,258 m to 2,178 m (passive) then in 3:30 h to 3,269 m (active) - Familiarization session, BCA at 1,258 m (D1 S1)	Section 1 2.1-2.3 2.5-2.10 Section 3 4.8	3 assessments: upon arrival at 3,269 m (D1 S2), morning of the second day (D2 S3) morning of the third day (D3 S4)/NA	DSST[1] (*attention*) PVT[1] (*reaction time*) Balloon Analogue Risk Task (BART) (*risky decision making*) + Hospital anxiety and depression scale, State Trait Anxiety Inventory, Pittsburgh Sleep Quality Index, Insomnia Severity Index, Perceived Stress Scale, Wagnild and Young's scale	<u>DSST</u> ↓ Number of correct responses (processing speed) (D2 S3), ↔ Back to normal (D3 S4) <u>PVT</u> ↔ Mean reaction time, number of lapses and number of false starts ↓ Higher number of false starts with higher Insomnia Severity Index <u>BART</u> ↑ Faster total time (D3 S4), ↑ Mean earnings (D3 S4) ↔ Number of pumps
Frost (2021) [43]	15 (5 F)/M 24.9 ± 4.3 y; F 26.4 ± 5.1 y (M+F range 19-32 y)	3 days, field study 340 m to 1216 m in 4 h and from 1,216 m to 3,800 m in 2 h (passive) - Some assessments had practice sessions, Group split in half with either	Section 1 2.1-2.3 2.5 Section 3 4.8	Testing on three consecutive days at 3,800 m (ALT1, ALT2, ALT3)/30 min	Cognition by Joggle Research[1]: PVT (*reaction time*) BART (*risky decision making*) DSST (*attention*)	↓ <u>PVT</u> Slower reaction times (ALT3) <u>BART</u> ↑ Faster reaction times (ALT2 + ALT3) ↔ Number of pumps

First authors	Number of participants (sex)/Mean age of participants (Mean ± SD); profession if mentioned	Study protocol: Time of exposure, experimental condition, altitude profile, maximum altitude reached (Mode of ascent – active/passive)<br>-<br>Study protocol baseline or control group (CG), altitude (test event if named)	STAR core parameters	Timing of neuropsychological test administration under exposure (test event if named)/Duration of Test Battery	Test Battery: Neuropsychological tests (*Cognitive Domain*)<br>+<br>Further investigations that are not part of this review	Changes in cognitive performance ↔↑↓ based on significant results, $p < 0.05$ (test event if named)
		pre-ascent BCA or post-descent CA at 340 m ASL			Line Orientation Task (visuospatial analytic ability)<br>N-Back Task (executive function)<br>Visual Object Learning Task (*Visual memory*)<br>Abstract Matching (Visuospatial analytic ability)<br>Motor Praxis Task (*psychomotor ability*)<br>+<br>Nocturnal actigraphy and polygraphy, Pittsburgh Sleep Quality Index, Stanford Sleepiness Scale, PROMIS Sleep Disturbance questionnaire	↔ DSST, Line Orientation Task, N-Back Task, Abstract Matching, Motor Praxis Task
Gibbons (2020) [44]	10 (M)/27 ± 11 y	16 ± 4 days, field study, from SL in 6 h to 4,330 m (passive)<br>-<br>Familiarization test, BCA at 344 m prior to expedition	Section 1 2.1, 2.2, 2.5<br>4.7-4.9	One assessment after 16 ± 4 days/~3 min	Pro-point and Anti-Point Tasks (*executive function*)	↔ No change in performance
Griva (2017) [50]	198 (60 % M)/44.5 ± 13.7 y;<br><br>CG: 25 (60 % M)/44.5 ± 14.1 y	11 days, field study, from 3,500 m up to 5,300 m (active)<br>-<br>BCA at 75 m ASL prior to expedition<br><br>CG at/or near SL, NA	Section 1 2.1-2.3<br>2.5<br>Section 3 4.8, 4.9	3 assessments with CA on either day 1 or day 2 after arrival at:<br>3,500 m,<br>at 5,300 m on day 11,<br>at 1,300 m on return	Trail Making Test Parts A[1] (*attention*) and B[1] (*executive function*)<br>Controlled Oral Word Association Test[1] (*executive function*)<br>Letter Cancellation Test (*attention*)<br>Stroop Test (*executive function*)<br>Grooved Pegboard (*psychomotor ability)*	↓ Attention, verbal abilities and executive functioning<br>↓ Memory and psychomotor function<br>↑ Cognitive decline greater amongst older people<br>↓ Cognitive function after descent

First authors	Number of participants (sex)/Mean age of participants (Mean ± SD); profession if mentioned	Study protocol: Time of exposure, experimental condition, altitude profile, maximum altitude reached (Mode of ascent – active/passive) - Study protocol baseline or control group (CG), altitude (test event if named)	STAR core parameters	Timing of neuropsychological test administration under exposure (test event if named)/Duration of Test Battery	Test Battery: Neuropsychological tests (*Cognitive Domain*) + Further investigations that are not part of this review	Changes in cognitive performance ↔↑↓ based on significant results, $p < 0.05$ (test event if named)
					Rey Auditory Verbal Learning Test (*verbal memory*)<br>Symbol Digit Modalities Test[1] (*attention*)<br>Block Design Test (*assembling and building*)<br>+<br>Brief Center for Epidemiologic Studies Depression Scale, State Trait Anxiety Inventory	
Harris (2009) [25]	26 (11 F)/M 34.9 (range 23-53) y; F 32.5 (range 25-40) y;<br><br>CG: 411 (32 F)/M 34.2 (range 25-55) y, F 34.2 (range 25-40) y	18 days, field study, from 410 m up to 5,400 m (active)<br>-<br>Practice test, CA at SL prior to expedition<br><br>CG, NA	Section 1 2.1-2.3	One assessment within the first 24 hours of arrival at 5,100 m/NA	DST-F[1] (*short term memory*)<br>DST-B[1] (*working memory*)<br>DSST[1] (*attention*)<br>Trail-making Test, Part B[1] (*executive function*)<br>Rey's Auditory-Verbal Learning Test[1] (*verbal memory*)<br>Controlled Oral Word Association Test[1] (*executive function*)<br>CogState[1]:<br>Simple Reaction Time (*reaction time*)<br>Choice Reaction Time (*executive function*)<br>Monitoring Task Reaction Time (*attention*)<br>Monitoring Task Accuracy (*attention*)<br>Working Memory Task Reaction Time (*attention*)<br>Working Memory Task Accuracy (*working memory*),<br>Learning Task Accuracy (*visual memory*)	↓ DST-F<br>↑ DSST<br>↑ Trail-Making Test, Part B<br>↑ CogState Monitoring Test improved reaction time<br><br>Performance was more variable in the written than the computerized tests

First authors	Number of participants (sex)/Mean age of participants (Mean ± SD); profession if mentioned	Study protocol: Time of exposure, experimental condition, altitude profile, maximum altitude reached (Mode of ascent – active/passive) - Study protocol baseline or control group (CG), altitude (test event if named)	STAR core parameters	Timing of neuropsychological test administration under exposure (test event if named)/Duration of Test Battery	Test Battery: Neuropsychological tests (*Cognitive Domain*) + Further investigations that are not part of this review	Changes in cognitive performance ↔↑↓ based on significant results, $p < 0.05$ (test event if named)
Issa (2016) [23]	8 (NA)/35 ± 10 y	23 days, field study, 1,400 m up to 5,500 m (active) - Familiarization trial, Pre-ascent BCA (Baseline) and post-descent CA (Post) at 1,400 m Retesting after return to USA	Section 1 2.1, 2.3 Section 3	3 assessments at 5,500 m on day 2 (T1), day 5 (T2) and day 7 (T3) after arrival at base camp/NA	Rapid Cognitive Assessment Tool (*executive function*) Stroop Color-Word test (*attention*) Trail Making test part A (*attention*) and B (*executive function*) + Profile of Mood States self-report questionnaire, Spielberger State-Trait Anxiety Inventory, Acceptance and Action Questionnaire-II	No calculations of significance but trend to improvement regarding baseline and base camp ↔ No significant differences between scores taken at base camp (T1, T2, T3) and the post expedition scores (Post) for all 3 tests. Stroop test performance worsening associated with increase in AMS
Karinen (2017) [40]	9 (M)/37.6 ± 5.5 (range 27–45) y; experienced climbers	80 days, field study, 1,400 m to 8,848 m (active) - Pre-ascent BCA (d2) and post-descent CA (d69) at 1,400 m	Section 1 2.1-2.3 2.10	4 assessments each at 5,300 m; the first 2 days after arrival at base camp (d16), then 2 days after setting up camp 3 at 7100 m (d34), the third after 4 days' rest (d43) and 4 days after successful summiting (d62)	Colorado Perceptual Speed Test[1] (*attention*) Number Comparison Test[1] (*executive function*)	↑ Colorado Perceptual Speed Test Improving speed results during the expedition ↔ Number Comparison Test
Kourtidou -Papadeli (2008) [41]	Group II: 10 (M+F; NA)/32.1 ± 6,74 y; some are private pilots	NA, breathing of hypoxic gas mixture via face mask equivalent to 2,440 m ≙ 8,000 ft - BCA under normoxia with two-week training	1.1-1.3 2.1, 2.2, 2.5 4.7-4.9	Time of hypoxia exposure NA/16 min	MATB – a PC based Multiple Attribute Task Battery[1] (*executive function*) multitask flight simulation package with monitoring, tracking,	↓ Decrease in performance with statistically significant increase of Root mean scare error compared to normoxic and hyperoxic condition

First authors	Number of participants (sex)/Mean age of participants (Mean ± SD); profession if mentioned	Study protocol: Time of exposure, experimental condition, altitude profile, maximum altitude reached (Mode of ascent – active/passive) - Study protocol baseline or control group (CG), altitude (test event if named)	STAR core parameters	Timing of neuropsychological test administration under exposure (test event if named)/Duration of Test Battery	Test Battery: Neuropsychological tests (*Cognitive Domain*) + Further investigations that are not part of this review	Changes in cognitive performance ↔↑↓ based on significant results, $p < 0.05$ (test event if named)
		to establish a personal performance plateau, post exposure CA under 100 % O_2			communications, and fuel management tasks<br>+<br>EEG measurements	
Kramer (1993) [28]	20 (2 F)/31.2 ± 5.2 y, APTS climbers; 32.3 ± 4.6, category search climbers<br><br>CG: 20 (2 F)/29.3 ± 4.8 y, APTS control; 30.9 ± 3.8 y category search control	Range from 18 to 26 days, field study, from 2,195 m up to NA (active)<br>-<br>Pre-ascent BCA and post-descent CA at 92 m<br><br>CG, NA	Section 1 2.1, 2.2 3.1-3.6	Testing altitudes NA/NA	<u>1st group + CG</u>: Automated Performance Test System (APTS) battery: Pattern Comparison Task (*visuospatial analytic ability*), Code (Letter Number) Substitution Task (*attention*) Choice Reaction Time Task (*executive function*) Memory Search Task (*short term memory*) Finger-Tapping Task (*psychomotor ability*) <u>2nd group + CG</u>: Category Search Task (*efficacy, reasoning*) Consistently Mapped Varied Mapping	↓ Slow performance with higher reaction and response times<br>↔ No improvement in performance compared to CG<br><br>Learning effects and outcome ↑ in the Consistently Mapped Task than in the Varied Mapping Task<br>↓ Levels of transfer compared to control
Latshang (2013) [38]	51 (M)/24 (quartiles 20-28) y	4 days, field study, from 490 m up to 1,630 m and 2,590 m (passive) four groups with block designed altitude exposure<br>-<br>Pre-ascent BCA and Post-descent CA at 490 m	Section 1 2.1-2.3 2.5, 2.6 Sections 3 & 4	4 assessments, 2x after night at 1,630 m an 2x at 2,590 m in randomized order/NA	PVT (*reaction time*) Divided Attention Steering Simulator (*attention*) 1-, 2-, and 3-Number Back Task (*executive function*) Trail Making Test A (*attention*)	↔ No change in performance detected
Lefferts (2019) [51]	18 (8 F)/ M 32 ± 15 y; F 20 ± 1 y; (M + F range 18–60) y	11 days, field study, 1,400 m up to 5,160 m (active)<br>-<br>Extensive familiarization process prior to the	Section 1 2.1, 2.2, 2.5 Section 3 4.7, 4.8	3 assessments: day 4 at 3,440 m, day 8 at 4,240 m;	Flanker Task (attention) 2-Back version of an N-Back Number Task (executive function)	<u>Flanker Task</u><br>↔ Accuracy<br>↑ Faster RT at 4,240 m<br><u>N-Back Task</u><br>↓ Decreased accuracy at 5,160 m

First authors	Number of participants (sex)/Mean age of participants (Mean ± SD); profession if mentioned	Study protocol: Time of exposure, experimental condition, altitude profile, maximum altitude reached (Mode of ascent – active/passive) - Study protocol baseline or control group (CG), altitude (test event if named)	STAR core parameters	Timing of neuropsychological test administration under exposure (test event if named)/Duration of Test Battery	Test Battery: Neuropsychological tests (*Cognitive Domain*) + Further investigations that are not part of this review	Changes in cognitive performance ↔↑↓ based on significant results, $p < 0.05$ (test event if named)
		trek, and practice tests throughout the trek (hope of practice effects plateau);<br>CA prior to expedition at 116 m ASL		and day 11 at 5,160 m/NA		↑ Faster RT for 2-back at all altitudes<br>↓ Modest reductions in caution and non-decision time<br>↑ Bias and strength of evidence for non-match items during the 2-back
Lefferts (2020) [52]	8 (4 F)/23–7 (range 18–41) y	7-days, field study, 1,400 m up to 4,240 m (active)<br>-<br>BCA on day 0 at 1,400 m altitude	Section 1 2.1, 2.2, 2.5<br>Section 3 4.7, 4.8	2 assessments: day 3 at 3,440 and day 7 at 4,240 m/3 min	Stroop Task (*Executive function*)	↑ Accuracy at 4,240 m<br>↑ Faster reaction times at 3,440 m and 4,240 m
Limmer (2018) [36]	80 (29 F)/M 25.5 ± 6.0 y; F 24.8 ± 5.9 y<br><br>4 groups<br><u>HYP + EX (hypoxia + exercise)</u>: 15 (3 F)/ M 27.3 ± 11.6 y, F 32.7 ± 17.6 y;<br><u>HYP</u>: 25 (6 F)/M 24.7 ± 3.1 y, F 22.7 ± 2.3 y;<br><u>NOR-normoxia</u>: 21 (10 F)/M 24.7 ± 3.1 y, F 24.4 ± 2.2 y;<br><u>EX</u>: 21 (10 F)/M 24.4 ±	<u>HYP + EX:</u> 7-days, field study, starting altitude NA up to 5,739 m (active)<br>-<br>CA prior to expedition at 154 m (D1) and post-descent CA at 812 m (D18)<br><br><u>HYP:</u> 2x21 minutes, normobaric chamber, 3,500 m ≙ F_iO_2 13,5 % O_2 (D14) and 5,800 m ≙ F_iO_2 10.0 % (D16)<br>-<br>Pre- (D1) and post-CA (D18) at 53 m normoxic conditions<br><br><u>NOR:</u> CG at normoxia<br><br><u>EX:</u> CG with 7-days physical exercise at normoxia	Section 1 2.1, 2.2, 2.5<br>Section 3	2 assessments on days 14 and 16:<br>HYP + EX: day 4 of expedition at 3,995 m (D14), day 6 of expedition at 5,739 m (D16)/6 min<br><br>HYP: day 14 after 15 min of exposure to hypoxia corresponding to 3,500 m (D14), day 16 after 15 min corresponding to 5,800 m (D16)/6 min	Frankfurt Attention Inventory-2 (*attention*) Performance Value, Continuity Value and Quality Value	HYP + EX:<br>↓ Attentional functions in Performance Value and Continuity Value at 5,739 m<br>↔ Quality Value<br><br>HYP:<br>↓ Attentional functions in Performance Value, Continuity Value and Quality Value at 5,800 m

First authors	Number of participants (sex)/Mean age of participants (Mean ± SD); profession if mentioned	Study protocol: Time of exposure, experimental condition, altitude profile, maximum altitude reached (Mode of ascent – active/passive) - Study protocol baseline or control group (CG), altitude (test event if named)	STAR core parameters	Timing of neuropsychological test administration under exposure (test event if named)/Duration of Test Battery	Test Battery: Neuropsychological tests (*Cognitive Domain*) + Further investigations that are not part of this review	Changes in cognitive performance ↔↑↓ based on significant results, $p < 0.05$ (test event if named)
	1.3 y, F 24.0 ± 2.3 y;					
Loprinzi (2019) [63]	21 (11 F)/21.0 (range 18-35) y	30 min, breathing of hypoxic gas mixture via face mask F_iO_2 = 12 % ≙ 4,000 m ASL - Approx. 24 h apart counterbalanced blinded condition with one control CA under normoxia	1.1-1.3 2.1-2.3, 2.5, 2.10 Section 3	After 30 min of exposure start of memory task assessment, after 20 min rest distraction assessment of delayed memory recall/NA	Memory Interference Task (AB/AC paradigm) (*working memory*) immediate and delayed proactive and retroactive interference	↔ Proactive interference ↑ Retroactive interference for both the immediate and delayed memory assessments, suggesting a reduced memory interference effect ≙ enhanced brain function
Malle (2016) [39]	4 (M)/29.2 ± 1.6 y; professional climbers	6-weeks, field study, from approx. 1,400 m start with 13-days preacclimatization trek up to 5,500 m, followed by a 4-week progressive ascent up to 8043 m (active) - Familiarization trial, 8-days (BL) prior to expedition and 4- (D61) and 46-days (D103) post expedition CA respectively at 1,050 m	Section 1 2.1-2.3 2.9 Section 3 4.8	14 assessments of PASAT between 1,400 m (D1) and 7,200 m (D40)/NA<br><br>6 assessments of DST subtests between 1,400 m (D1) and 5,600 m (D35)/NA	Paced auditory serial addition test (PASAT) (*attention*) DST-F (*short term memory*) and DST-B (*working memory*)	↔ No significant differences in any of the three cognitive tasks
Merz (2013) [53]	32 (7 F)/43 (range 25–62) y; experienced non-professional mountaineers	21-days until summit reach, field study, mean ascent rate 191-201 m/d, from 3,750 m up to 7,546 m (active) - Familiarization trial, Prior to (ZH1) and 3-months post-expedition CA (ZH2) at 440 m	Section 1 2.1, 2.2 Section 3 4.8	3 assessments: 4,497 m (BC), 5,533 m (C1), and at 6,265 m (C2)/NA	Saccadic Eye Movement (*Ocular motor performance*) Line Bisection Test (*visual perception*) Ruff 2/7 Cancellation Test – Letter and Number Condition[1] (*attention*)	↔ Eye movement experiments revealed no differences in either saccade latency or main sequence<br><br>↔ No impairments or dependence on any altitude-related parameter

First authors	Number of participants (sex)/Mean age of participants (Mean ± SD); profession if mentioned	Study protocol: Time of exposure, experimental condition, altitude profile, maximum altitude reached (Mode of ascent – active/passive) - Study protocol baseline or control group (CG), altitude (test event if named)	STAR core parameters	Timing of neuropsychological test administration under exposure (test event if named)/Duration of Test Battery	Test Battery: Neuropsychological tests (*Cognitive Domain*) + Further investigations that are not part of this review	Changes in cognitive performance ↔↑↓ based on significant results, $p < 0.05$ (test event if named)
					Ruff Figural Fluency Test (RFFT) (*executive function*)<br>Modified Pegboard, Chess test (*Psychomotor ability*)	
Nakano (2015) [77]	7 (1 F)/NA (range 19–46 y); climbers	4 h 15 min, hypobaric chamber, barometric pressure decreases -2 mmHg/min ≙ ascent speed 33 m/min with maintained altitude conditions of 2,000 m, 3,000 m, 4,000 m, and 4,500 m for 30 min<br>-<br>No control condition, CA as widely adopted method with cut-off score set at 23/24	1.1-1.5<br>2.1-2.3<br>4.8-4.9	One assessment at 4,500 m ASL/NA	Mini-Mental State Examination (*concentration or working memory, language and prax-is, orientation, memory, attention span, and other cognitive factors*)	↔ Normal score of 29.3 ± 0.8.
Nelson (1990) [54]	12 (3 F)/NA; climbers	Duration NA, field test, from 1,200 m up to 8,848 m (active)<br>-<br>Pre-ascent (1) and approx. 1-week post-descent BCA (6) at 1,200 m	Section 1<br>2.2, 2.3	4 assessments: after 48 hrs at 5,400 m (2); 2 week trek to 6,500 m, after 48 hrs (3); approx. 2 weeks later at 6,500 m or 7,100 m (4); and at 5,400 m after having reaching ones highest point during this expedition (5)/NA	Modified version of the FACTRETRIEVAL2 test battery[1] (*long-term memory*)<br>Feeling of Knowing (*confidence-judgement*)	↔ <u>FACTRETRIEVAL2 test battery</u><br>No reliable changes in mean percent or latency of correct recall, no changes in mean percent correct recognition on nonrecalled items across the five testing locations<br><br>↓ <u>Feeling of Knowing</u> declined reliably between the first two tests and the last three tests, the decrease is still present more than a week later

First authors	Number of participants (sex)/Mean age of participants (Mean ± SD); profession if mentioned	Study protocol: Time of exposure, experimental condition, altitude profile, maximum altitude reached (Mode of ascent – active/passive)<br>-<br>Study protocol baseline or control group (CG), altitude (test event if named)	STAR core parameters	Timing of neuropsychological test administration under exposure (test event if named)/Duration of Test Battery	Test Battery: Neuropsychological tests (*Cognitive Domain*)<br>+<br>Further investigations that are not part of this review	Changes in cognitive performance ↔↑↓ based on significant results, $p < 0.05$ (test event if named)
Niedermeier (2017) [67]	22 (11 F)/25.8 ± 5.5 y<br><br>CG: 20 (8 F)/24.8 ± 4.6 y	12 h, normobaric chamber<br>F_iO_2 = 12.6 % ≙ 4500 m after short-term pre-acclimatization using intermittent hypoxia 7 × 1 h F_iO_2 12.6 % (without physical activity)<br>-<br>Familiarization session CG with sham pre-acclimatization 7 × 1 h at F_iO_2 = 20.9 % ≙ 600 m ASL	1.1-1.5<br>2.1-2.3<br>Section 3<br>4.8	4 data collection points after 2, 5, 8, and 10 h in hypoxia/NA	Computer-based Game of Dice Task[1] (*Risky decision making*)	↓ Adverse risk behavior at 4,500 m<br>But reduced risk behavior in preacclimatized subjects compared to CG<br><br>Positive time effect on decision making: independent of the group effect, the number of risky decisions decreased over time in hypoxia after controlling for the covariate age.
Ochi (2018) [64]	21 (7 F)/20.5 ± 2.5 (range 18–29) y	16,5 min, breathing of hypoxic gas mixture via face mask F_iO_2 = 16,5 (mild)/13,5 (moderate)/10,5 (severe) % ≙ 2,000/3,500/5,000 m ASL<br><br>Two practice sessions, CA at SL	1.1-1.3<br>2.1, 2.2<br>4.7-4.9	2 d with 2 conditions per day in randomized order, >48 h between first and second day; assessment after 10-min exposure/6,5 min	Color–Word Stroop Task (*executive function*)	↓ Slower reaction times at 5,000 m<br>↔ Error rate<br>Significant main effect of condition for reaction time in Stroop interference<br>Negative correlation between reaction time in Stroop interference and S_pO_2
Parker (2017) [68]	10 (3 F)/NA; orthopedic surgeons	45 min, normobaric environmental chamber, F_iO_2 = 14.1 % ≙ 3,000 m ≙ 10,000 ft ASL<br>-<br>Practice trial before VWMC, BCA before entering the chamber; Double blind, repeated CA under normobaric normoxia F_iO_2 = 20.9 % near SL ≈ 113 m	1.1-1.4<br>2.2<br>4.7-4.9	After 15 min of exposure first VWMC assessment/4-10 min; then surgical skill/maximum of 15 min; then again VWMC assessment	Operational Span protocol - Verbal Working Memory Capacity (VWMC) with counting span, operation span, and reading span (*working memory*)<br>Surgical Skills by application of an orthopedic external fixator (*psychomotor ability*)	↔ <u>VWMC</u><br><u>Surgical Skills</u><br>↔ Completion time<br>↓ Worse application with higher pin-divergence score as a measure of frame asymmetry

First authors	Number of participants (sex)/Mean age of participants (Mean ± SD); profession if mentioned	Study protocol: Time of exposure, experimental condition, altitude profile, maximum altitude reached (Mode of ascent – active/passive)<br>-<br>Study protocol baseline or control group (CG), altitude (test event if named)	STAR core parameters	Timing of neuropsychological test administration under exposure (test event if named)/Duration of Test Battery	Test Battery: Neuropsychological tests (*Cognitive Domain*)<br>+<br>Further investigations that are not part of this review	Changes in cognitive performance ↔↑↓ based on significant results, $p < 0.05$ (test event if named)
Pavlicek (2005) [78]	21 (M)/24.2 ± 2.4 y<br><br>AP1/AP2/CP group with 7 subjects each	2 h, hypobaric chamber,<br>• Altitude profile 1 (AP1):<br>450 (TS1); 1,500 (TS2); 4,500 (TS3) m<br>• Altitude profile 2 (AP2):<br>450 (TS1); 1,500 (TS2); 3,000 (TS3) m<br>-<br>• Control profile (CP): 450 (TS1); 650 (TS2); 650 (TS3) m with 200 m “pseudo-ascent” to mask for altitude	1.1-1.5<br>2.1-2.3<br>4.8, 4.9	three 30-min test sessions (TSs) separated by 10-min intervals for pressure adjustment/30 min, Letter fluency test 2 min; word association task 1 min	4 Word-Generation Fluency Tasks - one Verbal Letter Fluency Task and three Category Fluency Tasks (*executive function*)<br>Lateralized Tachistoscopic Lexical Decision Task with high and low emotional target words (*affective flexibility*)	↔ No significant differences in word fluency, word association, or lateralized lexical decision performances
Pelamatti (2003) [55]	15 (NA)/34.5 (range 29-37) y; mountaineers	3-5 days, field study, 400 m/day, up to an altitude between 4,500 m-5,050 m during different expeditions (active)<br>-<br>Pre-BCA, twice 15- and 40-days Post-CAs	1.5, 1.6<br>2.1	Assessment at 4,500 m respectively 5,050 m/NA	Verbal Free Recall: proper vs. common names (*short-term memory*)	↓ Recall of proper names<br>↓ Primacy effect<br>↔ Recency effect
Petiet (1988) [56]	8 (F)/33.8 ± 3.8 (range 29-40) y; climbers	40-days, field study, maximum altitude ranged from 17,300 ft ≙ 5,273 m to 20,500 ft ≙ 6,248 m (active)<br>-<br>CA prior to expedition at SL and BCA on Day 6 at 4,000 ft ≙ 1,219 m<br>Post expedition CA at SL on between 16 to 221 days following the hypoxic exposure	Section 1<br>2.1, 2.2, 2.6-2.8<br>5.1	3 assessments:<br>Day 13 at 12,000 ft. ≙ 3,658 m;<br>Day 17 at 14,800 ft. ≙ 4,511 m;<br>Day 31.4 ± 12 (range 30-41 d) at highest elevation ranging from 5,273- 6,348 m/NA	Gorham's Proverbs (*Concept formation*)<br>PASAT (*attention*)<br>DST (*short term memory*)<br>Finger Tapping (*psychomotor ability*)<br>Selective Auditory Attention Task (*attention*)<br>Selective Reminding Test (*verbal memory*)<br>Benton Line Orientation Task (*visuospatial analytic ability*)<br>Boston Naming Test[1] (*Speech production and syntax comprehension*)<br>+	↑ <u>PASAT</u>, also improved post expedition<br><br>↓ Expressive language ability, measured by the <u>Boston Naming Test</u>

First authors	Number of participants (sex)/Mean age of participants (Mean ± SD); profession if mentioned	Study protocol: Time of exposure, experimental condition, altitude profile, maximum altitude reached (Mode of ascent – active/passive) - Study protocol baseline or control group (CG), altitude (test event if named)	STAR core parameters	Timing of neuropsychological test administration under exposure (test event if named)/Duration of Test Battery	Test Battery: Neuropsychological tests (*Cognitive Domain*) + Further investigations that are not part of this review	Changes in cognitive performance ↔↑↓ based on significant results, $p < 0.05$ (test event if named)
					Self-perception inventory of cognitive and affective functioning; the Acceptance of Others Scale, Multiple Affect Adjective Checklist-Revised, Environmental Symptoms Questionnaire	
Phillips (1963) [57]	Experiment I: 5 (M)/range 18-20 y<br><br>Experiment II: 8 (M)/range 18-20 y	Experiment I:<br>3 days, field study, up to 12,470 ft ≙ 3,800 m ≙ 490 mm Hg (NA)<br>-<br>15-days familiarization with tests 300 ft ≙ 91 m ≙ 752 mm Hg<br><br>Experiment II:<br>2 days, field study up to 14,250 ft ≙ 4,340 m ≙ 455 mm Hg<br>-<br>Two groups, counterbalanced BCA at SL	1.1-1.3<br>2.1, 2.2<br>4.7, 4.8	Experiment I:<br>3 assessment days at 3,800 m/Rhymes and Numbers tests max. 60 sec each<br><br>Experiment II:<br>2 assessment days at 4,340 m	DST-F (*short term memory*)<br>Word Span Forward (*short term memory*)<br>Robinson's Rhymes and Numbers tests (*mental efficacy, reasoning*)	Experiment I at 3,800 m<br>↔ No changes<br><br>Experiment II at 4,340 m<br>↓ <u>Rhymes Test</u><br>↑ <u>Numbers Test</u>
Phillips (1966) [58]	18 (M)/NA	2-days, field study, up to 3,800 m (NA)<br>-<br>Two groups, counterbalanced BCA at SL	1.1-1.3<br>2.2, 2.3<br>4.7, 4.8	2 assessment days at 3,800 m/distraction task 30 sec	Word Span Forward - immediate and short-term recall with or without 30 sec distraction task of backward subtraction (*short term memory*)	↔ No significant changes in altitude<br>Only tendency for recall impairment with retention loss: forgetting more items at the end of a list
Pighin (2019) [69]	26 (14 F)/23.3 ± 6.8 y	Exact duration NA, normobaric chamber, F_iO_2 = 14.1 % ≙ 3,000 m ASL<br>-<br>Familiarization session, CA under normoxia F_iO_2 = 20.9 % ≙ 0 m ASL in randomized order	1.1-1.3<br>2.1, 2.2<br>4.8, 4.9	After 20 min of exposure/NA	BART (*Risky decision making*)	<u>BART</u><br>↓ Adverse risk-taking behaviour, higher number of pumps

First authors	Number of participants (sex)/Mean age of participants (Mean ± SD); profession if mentioned	Study protocol: Time of exposure, experimental condition, altitude profile, maximum altitude reached (Mode of ascent – active/passive) - Study protocol baseline or control group (CG), altitude (test event if named)	STAR core parameters	Timing of neuropsychological test administration under exposure (test event if named)/Duration of Test Battery	Test Battery: Neuropsychological tests (*Cognitive Domain*) + Further investigations that are not part of this review	Changes in cognitive performance ↔↑↓ based on significant results, $p < 0.05$ (test event if named)
		with experimental session				
Pighin (2012) [70]	30 (16 F)/M 23.3 ± 4 y, F 20.5 ± 1.9 y	70 minutes, normobaric chamber, F_iO_2 = 14.1 % ≙ 3,000 m ASL<br>-<br>Familiarization session, CA under normoxia F_iO_2 = 20.9 % ≙ 0 m ASL in randomized order with experimental session	1.1-1.3<br>2.1, 2.2<br>4.8, 4.9	After 25 min of exposure/45 min	Computer-based Psychomotor Speed Task (*psychomotor ability*)<br>Risk-Taking Task (*Risky decision making*)	↓ Psychomotor Speed Task<br>8,85 % slower reaction times<br>Risk-Taking Task<br>↓ Adverse risk-taking behaviour for choices involving losses<br>↔ Risk taking behaviour for choices involving gains
Pramsohler (2017) [71]	11 (5 F)/21 ± 2.1 y	Two separate nights, normobaric chamber<br>1st night F_iO_2 = 14,29 % ≙ 3,500 m ASL<br>2nd night F_iO_2 = 11,05 % ≙ 5,500 m<br>-<br>BCA at 450 m ≙ F_iO_2 = 20.93 %	1.1-1.5<br>2.1-2.3<br>Section 3	2 assessments directly after awakening; mean individual sleep duration: 1st night 7,5 h 2nd night 3,23 h/10 min	Choice reaction test (Schuhfried) (*executive function*)<br>+<br>Polysomnography	↓ Slower cognitive reaction times at 3,500 m and 5,500 m<br>→ Positive correlation ($r = 0,78$) with S_pO_2; lower S_pO_2 surprisingly correlated significantly with shorter cognitive reaction<br>↔ Motoric reaction time
Pun, Guadagni (2018) [31]<br><br>*(also see Pun, Hartmann (2018)* [32])	21 (13 F)/25.3 ± 3.8 y<br><br>CG: 17 (12 F)/24.9 ± 2.6 y)	2 x 6-days field study, Cycle 1: day exposition to 5,050 m, sleeping height 2,900 m (passive); Week of rest at 520 m; Cycle 2: re-exposition following identical schedule<br>-<br>Familiarization trial, 2x BCA and post-expedition CA at 502 m<br>CG at 1,103 m	Section 1<br>2.1, 2.2, 2.4<br>Section 3	2 assessments each cycle: acute day 1 (Cycle 1 HA1, Cycle 2 HA1), acclimatization day 6 (Cycle 1 HA6, Cycle 2 HA6)/30 min	CANTAB[1]:<br>Reaction Time Task (*reaction time*),<br>Attention Switching Task (*attention*),<br>Rapid Visual Processing (*attention*)<br>One Touch Stockings of Cambridge Task (*executive function*)	↑ Selective and sustained attention improves with acclimatization<br><br>↔ Improvement gained in cognitive functions during the acclimatization period in Cycle 1 did not carry over to the repeated exposure in Cycle 2
Pun, Hartmann (2018) [32]	21 (13 F)/25.3 ± 3.8 y	2 x 6-days field study, Cycle 1: day exposition to 5,050 m, sleeping height 2,900 m (passive);	Section 1<br>2.1, 2.2, 2.4<br>Section 3	2 assessments each cycle: acute day 1 (Cycle	PVT (*reaction speed*)<br>Trail Making Tests A & B (*attention & executive function*)	PVT<br>↓ Slower reaction times at acute exposure to 5,050 m

First authors	Number of participants (sex)/Mean age of participants (Mean ± SD); profession if mentioned	Study protocol: Time of exposure, experimental condition, altitude profile, maximum altitude reached (Mode of ascent – active/passive) - Study protocol baseline or control group (CG), altitude (test event if named)	STAR core parameters	Timing of neuropsychological test administration under exposure (test event if named)/Duration of Test Battery	Test Battery: Neuropsychological tests (*Cognitive Domain*) + Further investigations that are not part of this review	Changes in cognitive performance ↔↑↓ based on significant results, $p < 0.05$ (test event if named)
(also see Pun, Guadagni (2018) [31])		Week of rest at 520 m; Cycle 2: re-exposition following identical schedule - Familiarization trial, 2x BCA and post-expedition CA at 502 m		1 HA1, Cycle 2 HA1), acclimatization day 6 (Cycle 1 HA6, Cycle 2 HA6)/10 min	+ Actigraphy for Sleep Monitoring, Environmental Symptom Questionnaire, Handgrip strength	↔ Reaction speed recovers after 6 days at altitude and prevents impairments during subsequent altitude re-exposure <u>Trail Making Tests</u> ↔ reaction times ↑ further HA stay leads to improvement
Roach (2014) [33] *(also see Subudhi (2014) [34]*)	21 (9 F)/20.8 (range 19–23) y	16-days, field study, from 1,525 m in 3 h with supplemental oxygen up to 5,260 m (passive), after first assessment down to 3800 m for 4-days, then back up to 5,260 m - 30-days pior to (SL) and 3 months post-expedition CA at SL	Section 1 2.1, 2.2 5.2	2 assessments: Day 1 after arrival (ALT1) and day 16 (ALT16) at 5,260 m /NA, DANA 20 min	2x Simple Reaction Time (SRT) Test before (SRT1) and after (SRT2) completion of a 20-min DANA Test Battery; Change score dSRT = SRT1 minus SRT2 (*attention / mental fatigue)*	↓ Marked decrease in throughput <u>dSRT</u> with acute altitude exposure ↔ <u>dSRT</u> normalized with acclimatization
Schlaepfer (1992) [37]	10 (4 F)/23.8 ± 1.2 y	25 min, 1. field study, from 540 m in <10 min up to 3,450 m (passive) 2. Breathing of hypoxic gas mixture via face mask F_iO_2 = 14.5 % ≙ 3,450 m - Familiarization trial, BCA at 540 m	Section 1 2.1, 2.2 2.10	Respective assessments within 15 min after exposure/10 min	Time Needed Reading Briefly Displayed Letters (*Visual perception*)	↑ Faster completion time in both hypoxic conditions
Seo (2015) [72]	16 (M)/24 ± 4 y	2 h, normobaric chamber, F_iO_2 = 12.5 % ≙ 4,300 m ≙ 14,110 ft ASL - Familiarization trial and BCA during rest in normoxia	1.1-1.3 2.1-2.3, 2.5 4.8, 4.9	One assessment after 60-min at rest/5 min	ANAM -4th Edition subtests[1]: Go/No-Go Test (*response inhibition*) Running Memory Continuous Performance	↔ <u>Go/No-Go Test</u> no statistical differences ↓ <u>RMCPT</u> less % correct and throughput score impaired compared with rest in normoxia

First authors	Number of participants (sex)/Mean age of participants (Mean ± SD); profession if mentioned	Study protocol: Time of exposure, experimental condition, altitude profile, maximum altitude reached (Mode of ascent – active/passive) - Study protocol baseline or control group (CG), altitude (test event if named)	STAR core parameters	Timing of neuropsychological test administration under exposure (test event if named)/Duration of Test Battery	Test Battery: Neuropsychological tests (*Cognitive Domain*) + Further investigations that are not part of this review	Changes in cognitive performance ↔↑↓ based on significant results, $p < 0.05$ (test event if named)
		+ Further examination but not part of this review: CA under low and moderate exercise performance			Test (RMCPT) (*working memory*)	
Seo (2017) [73]	15 (F)/22 ± 2 y	2 h, normobaric chamber, F_iO_2 = 12.5 % ≙ 4,300 m ≙ 14,110 ft ASL - Familiarization trial and BCA during rest in normoxia F_iO_2 = 21 % + Further examination but not part of this review: CA under low and moderate exercise performance	1.1-1.3 2.1-2.3, 2.5 4.8, 4.9	Two assessments after 30- and after 60-min at rest/5 min	ANAM-4th Edition[1]: RMCPT (*working memory*) Total Mood Disturbance (*affective flexibility*)	↔ RMCPT no change after 30- or 60-min rest in hypoxia ↓ Total Mood Disturbance score worse after 30- and after 60-min rest in hypoxia compared to baseline
Shi (2016) [45]	30 (M)/25.2 ± 1.9 y	3 h, field study, up to 4,280 m (passive) Retrograde calculated division into AMS and non-AMS group - BCA "in the plain", altitude NA	Section 1 2.1-2.3 2.10 Section 3 4.8	One assessment after 3h of exposure/NA	Visual-DST (V-DST) (*short term memory*) Auditory-DST (A-DST) (*short term memory*) Paced Visual Serial Addition Test (PVSAT) (*attention*) PASAT (*attention*) Picture Recall Test (*short term memory*) Picture Recognition Test (*short term memory*)	↓ A-DST, PVSAT, PASAT, and Picture Recognition Test ↔ V-DST and Picture Recall Test AMS vs. non-AMS group: ↓ V-DST, A-DST, PVSAT, PASAT, and Picture Recognition Test scores ↔ Picture Recall Test scores not different between groups
Stepanek (2013) [65]	25 (11 F)/32.4 ± 9.8 y	5 min, Breathing of hypoxic gas mixture via face mask F_iO_2 = 8 % ≙ partial pressure 24.3 mmHg ≙ 23,300 ft ≙ 7,101 m	1.2, 1.3 2.1, 2.2, 2.5 Section 3	One assessment after 3 min exposure/<2 min	King-Devick Test (*ocular motor performance*)	↓ 18 % slower completion time during hypoxia compared to baseline test ↑ faster completion time upon returning to

First authors	Number of participants (sex)/Mean age of participants (Mean ± SD); profession if mentioned	Study protocol: Time of exposure, experimental condition, altitude profile, maximum altitude reached (Mode of ascent – active/passive) - Study protocol baseline or control group (CG), altitude (test event if named)	STAR core parameters	Timing of neuropsychological test administration under exposure (test event if named)/Duration of Test Battery	Test Battery: Neuropsychological tests (*Cognitive Domain*) + Further investigations that are not part of this review	Changes in cognitive performance ↔↑↓ based on significant results, $p < 0.05$ (test event if named)
		- Familiarization tests, BCA and 3-min post CA at normoxia				normoxia compared to hypoxic testing ↔ no difference between BCA and post CA
Subudhi (2014) [34] *(also see Roach (2014) [33])*	21 (9 F)/20.8 ± 1.4 POST7 group: 14 POST21 group: 7	16-days, field study, from 1,525 m in 3 h with supplemental oxygen up to 5,260 m (passive), after first assessment down to 3800 m for 3-nights, then back up to 5,260 m until day 16; Thereafter descent to 1,525 m for either 7- or 21-days (POST7 or POST21) with return and retesting at 5260 m - Pre-BCA (SL) 30-days pior to expedition at 130 m	Section 1 2.1-2.3, 2.5, Section 3 5.2	4 assessments: Day 1 after arrival (ALT1) and day 16 (ALT16) at 5,260 m; Retesting at 5,260 m after pausing either 7- or 21-days (POST7 or POST21)/NA	DANA[1]: SRT1 & SRT2 (*reaction time*), Procedural Reaction Time (*reaction time*), Go/No-Go (*response inhibition*), Code (Digit symbol) Substitution - simultaneous (*attention*) Code (Digit symbol) Substitution - delayed recall (*short term memory*) Spatial Discrimination (*visuospatial analytic ability*), Match to Sample (*visual memory*), Sternberg's Memory Search (*working memory*)	↓ 5/9 tests from SL to ALT1 and ↔ normalized back to SL values by ALT16 SRT Tests, Code Substitution - simultaneous, Match to Sample, Procedural Reaction Time<br><br>↔ 4/9 tests from SL to ALT1: Code Substitution - delayed recall, Spatial Discrimination, Go/No-Go, Memory Search<br><br>POST7 ↔ 2/9 tests retention of acclimatization: Code Substitution – simultaneous, Match to Sample ↓ returning to ALT1 values indicating a loss of improvement in tests of reaction time (SRT Tests, Procedural Reaction Time)<br><br>POST21 ↓ No cognitive function tests showed retention of acclimatization
Turner (2015) [74]	22 (12 F)/23 ± 2 (range 20–28) y	90 min, Breathing of hypoxic gas mixture via face mask F_iO_2 = 10 % ≙ 5,500 m	1.2-1.3 2.1, 2.2, 2.5 3.1-3.6	One assessment after 50 min of breathing the	CNS Vital Signs[1]: Verbal and Visual Memory (*verbal memory, visual memory*)	↓ Hypoxia group: effect of hypoxia was detected for all cognitive domains: neurocognitive

First authors	Number of participants (sex)/Mean age of participants (Mean ± SD); profession if mentioned	Study protocol: Time of exposure, experimental condition, altitude profile, maximum altitude reached (Mode of ascent – active/passive) - Study protocol baseline or control group (CG), altitude (test event if named)	STAR core parameters	Timing of neuropsychological test administration under exposure (test event if named)/Duration of Test Battery	Test Battery: Neuropsychological tests (*Cognitive Domain*) + Further investigations that are not part of this review	Changes in cognitive performance ↔↑↓ based on significant results, $p < 0.05$ (test event if named)
		- BCA under normoxia Matched-pairs study with a single-blind, randomised design sham group F_iO_2 = 21 % ≙ SL	4.8, 4.9	gas mixture/40 min	Finger Tapping (*psychomotor ability*) Symbol digit coding (*attention*) Stroop Test (*executive function*) Shifting Attention (*executive function*) CPT (*attention*) + Environmental Symptoms Questionnaire	index (-20 %), composite memory (-30 %), verbal memory (-34 %), visual memory (-23 %), processing speed (-36 %), executive function (-20 %), psychomotor speed (-24 %), reaction time (-10 %), complex attention (-19 %) and cognitive flexibility (-18 %; all $p < 0.05$); No practice effects<br><br>↑ Sham group: Practice effects for information processing speed (+30 %), executive function (+14 %), psychomotor speed (+18 %), reaction time (+5 %), cognitive flexibility (+14 %), overall cognitive functioning (+9 %; all $p < 0.05$)
Weigle (2007) [59]	19 (9 F)/M 23.0 ± 3.0 y, F 21.4 ± 0.9 y	12 days, field study, exposition to altitudes between 4,000 – 14,250 ft ≙ 1,200 – 4,300 m (NA) - BCA at SL	1.1-1.5 2.1-2.3 Section 3 4.8	Two assessments at 3,810 m on day 3 and day 6/NA	Visual Motor Reaction Time (*psychomotor ability*) Stroop Color-Word Test (*executive function*) Verbal Reasoning Test: verbally presented problem set needing simple mathematical, spatial, and verbal analytic skills (*mental efficacy, reasoning*) Sentence Repetition (*short term memory*)	<u>Visual Motor Reaction Time</u> ↓ initially 3.2 % slower at 3,810 m, persisted with repeated measurements on day 6 m → impairment only seen in male subjects <u>Stroop Test</u> ↓ initially 27 % longer completion time at 3,810 m ↔ No longer persistent by day 6

First authors	Number of participants (sex)/Mean age of participants (Mean ± SD); profession if mentioned	Study protocol: Time of exposure, experimental condition, altitude profile, maximum altitude reached (Mode of ascent – active/passive) - Study protocol baseline or control group (CG), altitude (test event if named)	STAR core parameters	Timing of neuropsychological test administration under exposure (test event if named)/Duration of Test Battery	Test Battery: Neuropsychological tests (*Cognitive Domain*) + Further investigations that are not part of this review	Changes in cognitive performance ↔↑↓ based on significant results, $p < 0.05$ (test event if named)
						↔ Verbal Reasoning Test and Sentence Repetition at 3,810 m
Williams (2019) [75]	11 (M)/22 ± 4 y	4 x 60 min in normobaric, environmental chamber, blinded to the condition, F_iO_2: 20,93 % ≙ SL; 17,0 % ≙ 1,600 m; 14,5 % ≙ 3,000 m; 12,0 % ≙ 4,500 m<br>-<br>Familiarization session; CA under SL conditions; BCA prior to exposure under supply of normoxic air	1.1-1.3<br>2.1-2.3, 2.5, 2.10<br>Section 3<br>4.2, 4.7, 4.8	Assessments respectively after 60 min of exposure/NA	Eriksen flanker (*attention*)<br>N-Back Number Task (*executive function*)<br>Deary–Liewald Reaction Time Task (*reaction time*)	↓ N-Back Task accuracy at F_iO_2 = 14,5 % compared to baseline, 20,93 % and 17,0 %; at F_iO_2 = 12,0 % compared to 20,93 % but not to baseline<br><br>↔ Eriksen flanker and Deary–Liewald Reaction Time Task performance
Zhang (2013) [60]	46 (M)/20.41 - 1.58 y<br><br>not part of this review: chronic exposure groups: 3 x 50 subjects each, individuals native to high altitudes of 3,700, 4,500 and 5,100 m	5 days, field study, 3,700 m (NA)<br>-<br>BCA at 300 m	1.3<br>2.1-2.5<br>2.10		Neurobehavioral core test battery approved by the WHO<br>Simple Reaction Time Test (*reaction time*),<br>DST-F and DST-B (*short term memory, working memory*),<br>Santa Ana Manual Dexterity Test (*psychomotor ability*),<br>DSST (*attention*),<br>Benton Visual Retention Test (*copying*),<br>Pursuit Aiming Test (*psychomotor ability*)<br>+<br>Mood state profile	↓ Santa Ana Manual Dexterity Test, DSST, and Pursuit Aiming Test values decreased after 5 days at 3,700 m compared to their previous SL performance<br><br>↔ No change in Simple Reaction Time, DST, and Benton Visual Retention Test

Legend.

x[1]	Alternate forms used	DST-B	Digit Span Test Backward
A-DST	Auditory Digit Span Test	F	Female
ASL	Above sea level	M	Male
BART	Balloon Analogue Risk Task	NA	Information not available
BCA	Baseline cognitive assessment	PASAT	Paced Auditory Serial Addition Test
CA	Cognitive assessment	PVSAT	Paced Visual Serial Addition Test
CG	Control group	PVT	Psychomotor Vigilance Test
CPT	Continuous Performance Test	RMCPT	Running Memory Continuous Performance Test
DANA	Defense Automated Neurobehavioral Assessment Test Battery	SL	Sea level
		S_pO_2	Simple Reaction Time
dSRT	Change score = SRT1 minus SRT2	SRT	Oxygen saturation
DSST	Digit Symbol Substitution Test	V-DST	Visual Digit Span Test
DST	Digit Span Test	VWMC	Verbal Working Memory Capacity
DST-F	Digit Span Test Forward		

Note.
For a better overview, only the first author and the year of publication are given in addition to the numerical source citation. The arrow symbols represent the results of subsequently mentioned tests with ↔ meaning no effects, unchanged performance, ↑ standing for increased or better performance and ↓ for reduced or worse performance. In Table S2 an overview of the study findings regarding the impact of altitude exposure on cognitive performance is shown. Studies are presented with number, sex and age of the participants, the study protocol with temporal duration, type of intervention, altitude profile and information on the control group/examination, numbers of the STAR core parameters if considered in the study, the temporal and situational use of the neuropsychological tests, and the neuropsychological tests with their cognitive domain.

References

23. Issa AN, Herman NM, Wentz RJ, Taylor BJ, Summerfield DC, Johnson BD. Association of Cognitive Performance with Time at Altitude, Sleep Quality, and Acute Mountain Sickness Symptoms. *Wilderness Environ Med*. 2016; *27(3)*:371-8.
24. Davranche K, Casini L, Arnal PJ, Rupp T, Perrey S, Verges S. Cognitive functions and cerebral oxygenation changes during acute and prolonged hypoxic exposure. *Physiol Behav*. 2016; *164(Pt A)*:189-97.
25. Harris GA, Cleland J, Collie A, McCrory P. Cognitive Assessment of a Trekking Expedition to 5100 m: A Comparison of Computerized and Written Testing Methods. *Wilderness Environ Med*. 2009; *20(3)*:261-8.
27. Asmaro D, Ferguson S, Mayall J. Cognition at altitude: Impairment in executive and memory processes under hypoxic conditions. *Aviat Space Environ Med*. 2013; *84(11)*:1159-65.
28. Kramer AF, Coyne JT, Strayer DL. Cognitive function at high altitude. *Hum Factors*. 1993; *35(2)*:329-44.
31. Pun M, Guadagni V, Bettauer KM, Drogos LL, Aitken J, Hartmann SE, et al. Effects on Cognitive Functioning of Acute, Subacute and Repeated Exposures to High Altitude. *Front Physiol*. 2018; *9*:1131.
32. Pun M, Hartmann SE, Furian M, Dyck AM, Muralt L, Lichtblau M, et al. Effect of Acute, Subacute, and Repeated Exposure to High Altitude (5050 m) on Psychomotor Vigilance. *Front Physiol*. 2018; *9*:677.
33. Roach EB, Bleiberg J, Lathan CE, Wolpert L, Tsao JW, Roach RC. AltitudeOmics: Decreased reaction time after high altitude cognitive testing is a sensitive metric of hypoxic impairment. *Neuroreport*. 2014; *25(11)*:814-8.
34. Subudhi AW, Bourdillon N, Bucher J, Davis C, Elliott JE, Eutermoster M, et al. AltitudeOmics: the integrative physiology of human acclimatization to hypobaric hypoxia and its retention upon reascent. *PloS One*. 2014; *9(3)*:e92191.
35. Brodmann Maeder M, Brugger H, Pun M, Strapazzon G, Dal Cappello T, Maggiorini M, et al. The STAR Data Reporting Guidelines for Clinical High Altitude Research. *High Alt Med Biol*. 2018; *19(1)*:7-14.
36. Limmer M, Platen P. The influence of hypoxia and prolonged exercise on attentional performance at high and extreme altitudes: A pilot study. *PloS One*. 2018; *13(10)*:e0205285.
37. Schlaepfer TE, Bartsch P, Fisch HU. Paradoxical effects of mild hypoxia and moderate altitude on human visual perception. *Clin Sci (Lond)*. 1992; *83(5)*:633-6.
38. Latshang TD, Lo Cascio CM, Stowhas AC, Grimm M, Stadelmann K, Tesler N, et al. Are nocturnal breathing, sleep, and cognitive performance impaired at moderate altitude (1,630-2,590 m)? *Sleep*. 2013; *36(12)*:1969-76.
39. Malle C, Ginon B, Bourrilhon C. Brief Working Memory and Physiological Monitoring During a High-Altitude Expedition. *High Alt Med Biol*. 2016; *17(4)*:359-64.
40. Karinen HM, Tuomisto MT. Performance, Mood, and Anxiety During a Climb of Mount Everest. *High Alt Med Biol*. 2017; *18(4)*:400-10.
41. Kourtidou-Papadeli C, Papadelis C, Koutsonikolas D, Boutzioukas S, Styliadis C, Guiba-Tziampiri O. High altitude cognitive performance and COPD interaction. *Hippokratia*. 2008; *12 Suppl 1*:84-90.
42. Abraini JH, Bouquet C, Joulia F, Nicolas M, Kriem B. Cognitive performance during a simulated climb of Mount Everest: implications for brain function and central adaptive processes under chronic hypoxic stress. *Pflügers Archiv*. 1998; *436(4)*:553-9.
43. Frost S, J EO, Oeung B, Puvvula N, Pham K, Brena R, et al. Improvements in sleep-disordered breathing during acclimatization to 3800 m and the impact on cognitive function. *Physiol Rep*. 2021; *9(9)*:e14827.

44. Gibbons TD, Tymko MM, Thomas KN, Wilson LC, Stembridge M, Caldwell HG, et al. Global REACH 2018: The influence of acute and chronic hypoxia on cerebral haemodynamics and related functional outcomes during cold and heat stress. *J Physiol*. 2020; *598(2)*:265-84.
45. Shi QH, Ge D, Zhao W, Ma X, Hu KY, Lu Y, et al. A Computerized Evaluation of Sensory Memory and Short-term Memory Impairment After Rapid Ascent to 4280 m. *Biomed Environ Sci*. 2016; *29(6)*:457-60.
46. Bjursten H, Ederoth P, Sigurdsson E, Gottfredsson M, Syk I, Einarsson O, et al. S100B profiles and cognitive function at high altitude. *High Alt Med Biol*. 2010; *11(1)*:31-8.
47. Bonnon M, Noel-Jorand MC, Therme P. Criteria for psychological adaptation to high-altitude hypoxia. *Percept Mot Skills*. 1999; *89(1)*:3-18.
48. Dykiert D, Hall D, van Gemeren N, Benson R, Der G, Starr JM, et al. The effects of high altitude on choice reaction time mean and intra-individual variability: Results of the Edinburgh Altitude Research Expedition of 2008. *Neuropsychology*. 2010; *24(3)*:391-401.
49. Falla M, Papagno C, Dal Cappello T, Vögele A, Hüfner K, Kim J, et al. A Prospective Evaluation of the Acute Effects of High Altitude on Cognitive and Physiological Functions in Lowlanders. *Front Physiol*. 2021; *12*:670278.
50. Griva K, Stygall J, Wilson MH, Martin D, Levett D, Mitchell K, et al. Caudwell Xtreme Everest: A prospective study of the effects of environmental hypoxia on cognitive functioning. *PloS One*. 2017; *12(3)*:e0174277.
51. Lefferts WK, DeBlois JP, White CN, Day TA, Heffernan KS, Brutsaert TD. Changes in cognitive function and latent processes of decision-making during incremental ascent to high altitude. *Physiol Behav*. 2019; *201*:139-45.
52. Lefferts WK, DeBlois JP, Soriano JE, Mann L, Rampuri Z, Herrington B, et al. Preservation of Neurovascular Coupling to Cognitive Activity in Anterior Cerebrovasculature During Incremental Ascent to High Altitude. *High Alt Med Biol*. 2020; *21(1)*:20-7.
53. Merz TM, Bosch MM, Barthelmes D, Pichler J, Hefti U, Schmitt K-U, et al. Cognitive performance in high-altitude climbers: a comparative study of saccadic eye movements and neuropsychological tests. *Eur J Appl Physiol*. 2013; *113(8)*:2025-37.
54. Nelson TO, Dunlosky J, White DM, Steinberg J, Townes BD, Anderson D. Cognition and metacognition at extreme altitudes on Mount Everest. *J Exp Psychol Gen*. 1990; *119(4)*:367-74.
55. Pelamatti G, Pascotto M, Semenza C. Verbal Free Recall in High Altitude: Proper Names vs Common Names. *Cortex*. 2003; *39(1)*:97-103.
56. Petiet CA, Townes BD, Brooks RJ, Kramer JH. Neurobehavioral and psychosocial functioning of women exposed to high altitude in mountaineering. *Percept Mot Skills*. 1988; *67(2)*:443-52.
57. Phillips LW, Griswold RL, Pace N. Cognitive changes at high altitude. *Psychol Rep*. 1963; *13(2)*:423-30E.
58. Phillips LW, Pace N. Performance changes at moderately high altitude: short-term memory measured by free recall. *Psychol Rep*. 1966; *19(2)*:655-65.
59. Weigle DS, Buben A, Burke CC, Carroll ND, Cook BM, Davis BS, et al. Adaptation to altitude as a vehicle for experiential learning of physiology by university undergraduates. *Adv Physiol Educ*. 2007; *31(3)*:270-8.
60. Zhang G, Zhou SM, Yuan C, Tian HJ, Li P, Gao YQ. The effects of short-term and long-term exposure to a high altitude hypoxic environment on neurobehavioral function. *High Alt Med Biol*. 2013; *14(4)*:338-41.
61. Altbacker A, Takacs E, Barkaszi I, Kormos T, Czigler I, Balazs L. Differential impact of acute hypoxia on event related potentials: impaired task-irrelevant, but preserved task-relevant processing and response inhibition. *Physiol Behav*. 2019; *206*:28-36.
62. Chroboczek M, Kostrzewa M, Micielska K, Grzywacz T, Laskowski R. Effect of Acute Normobaric Hypoxia Exposure on Executive Functions among Young Physically Active Males. *J Clin Med*. 2021; *10(8)*.
63. Loprinzi PD, Matalgah A, Crawford L, Yu JJ, Kong Z, Wang B, et al. Effects of Acute Normobaric Hypoxia on Memory Interference. *Brain Sci*. 2019; *9(11)*.
64. Ochi G, Kanazawa Y, Hyodo K, Suwabe K, Shimizu T, Fukuie T, et al. Hypoxia-induced lowered executive function depends on arterial oxygen desaturation. *J Physiol Sci*. 2018; *68(6)*:847-53.
65. Stepanek J, Cocco D, Pradhan GN, Smith BE, Bartlett J, Studer M, et al. Early detection of hypoxia-induced cognitive impairment using the King-Devick test. *Aviat Space Environ Med*. 2013; *84(10)*:1017-22.
66. de Aquino Lemos V, Antunes HK, dos Santos RV, Lira FS, Tufik S, de Mello MT. High altitude exposure impairs sleep patterns, mood, and cognitive functions. *Psychophysiology*. 2012; *49(9)*:1298-306.
67. Niedermeier M, Weisleitner A, Lamm C, Ledochowski L, Frühauf A, Wille M, et al. Is decision making in hypoxia affected by pre-acclimatisation? A randomized controlled trial. *Physiol Behav*. 2017; *173*:236-42.
68. Parker PJ, Manley AJ, Shand R, O'Hara JP, Mellor A. Working Memory Capacity and Surgical Performance While Exposed to Mild Hypoxic Hypoxemia. *Aerosp Med Hum Perform*. 2017; *88(10)*:918-23.
69. Pighin S, Bonini N, Hadjichristidis C, Schena F, Savadori L. Decision making under stress: mild hypoxia leads to increased risk-taking. *Stress*. 2019:1-8.
70. Pighin S, Bonini N, Savadori L, Hadjichristidis C, Antonetti T, Schena F. Decision making under hypoxia: Oxygen depletion increases risk seeking for losses but not for gains. *Judgm Decis Mak*. 2012; *7(4)*:472.
71. Pramsohler S, Wimmer S, Kopp M, Gatterer H, Faulhaber M, Burtscher M, et al. Normobaric hypoxia overnight impairs cognitive reaction time. *BMC Neurosci*. 2017; *18(1)*:43.
72. Seo Y, Burns K, Fennell C, Kim JH, Gunstad J, Glickman E, et al. The Influence of Exercise on Cognitive Performance in Normobaric Hypoxia. *High Alt Med Biol*. 2015; *16(4)*:298-305.
73. Seo Y, Gerhart HD, Stavres J, Fennell C, Draper S, Glickman EL. Normobaric Hypoxia and Submaximal Exercise Effects on Running Memory and Mood State in Women. *Aerosp Med Hum Perform*. 2017; *88(7)*:627-32.
74. Turner CE, Barker-Collo SL, Connell CJ, Gant N. Acute hypoxic gas breathing severely impairs cognition and task learning in humans. *Physiol Behav*. 2015; *142*:104-10.

75. Williams TB, Corbett J, McMorris T, Young JS, Dicks M, Ando S, et al. Cognitive performance is associated with cerebral oxygenation and peripheral oxygen saturation, but not plasma catecholamines, during graded normobaric hypoxia. *Exp Physiol*. 2019; *104(9)*:1384-97.
76. De Bels D, Pierrakos C, Bruneteau A, Reul F, Crevecoeur Q, Marrone N, et al. Variation of Cognitive Function During a Short Stay at Hypobaric Hypoxia Chamber (Altitude: 3842 M). *Front Physiol*. 2019; *10*:806.
77. Nakano T, Iwazaki M, Sasao G, Nagai A, Ebihara A, Iwamoto T, et al. Hypobaric hypoxia is not a direct dyspnogenic factor in healthy individuals at rest. *Respir Physiol Neurobiol*. 2015; *218*:28-31.
78. Pavlicek V, Schirlo C, Nebel A, Regard M, Koller EA, Brugger P. Cognitive and emotional processing at high altitude. *Aviat Space Environ Med*. 2005; *76(1)*:28-33.
